# Self- and Informant-Report Cognitive Decline Discordance and Mild Cognitive Impairment Diagnosis

**DOI:** 10.1001/jamanetworkopen.2025.5810

**Published:** 2025-04-18

**Authors:** Anna Aaronson, Adam Diaz, Miriam T. Ashford, Chengshi Jin, Rachana Tank, Melanie J. Miller, Jae Myeong Kang, Manchumad Manjavong, Bernard Landavazo, Joseph Eichenbaum, Diana Truran, Monica R. Camacho, Juliet Fockler, Derek Flenniken, Patrizia Vannini, Sarah Tomaszewski Farias, R. Scott Mackin, Michael W. Weiner, Rachel L. Nosheny

**Affiliations:** 1 Veterans Affairs Advanced Imaging Research Center, San Francisco Veteran’s Administration Medical Center, San Francisco, California; 2 Department of Radiology and Biomedical Imaging, University of California, San Francisco; 3 Northern California Institute for Research and Education, Department of Veterans Affairs Medical Center, San Francisco; 4 Department of Epidemiology and Biostatistics, University of California, San Francisco; 5 Dementia Research Centre, University College London Institute of Neurology, University College London, London, United Kingdom; 6 Department of Psychiatry, Gil Medical Center, Gachon University College of Medicine, Incheon, Republic of Korea; 7 Department of Psychiatry and Behavioral Sciences, University of California, San Francisco; 8 Division of Geriatric Medicine, Department of Internal Medicine, Faculty of Medicine, Khon Kaen University, Khon Kaen, Thailand; 9 Department of Neurology, Massachusetts General Hospital, Harvard Medical School, Boston; 10 Department of Neurology, Brigham and Women’s Hospital, Harvard Medical School, Boston, Massachusetts; 11 Department of Neurology, University of California, Davis, Sacramento; 12 Department of Medicine, University of California, San Francisco; 13 Department of Neurology, University of California, San Francisco

## Abstract

**Importance:**

Subjective report of cognitive and functional decline from participant–study partner dyads can efficiently assess risk of cognitive impairment and clinical progression. Accuracy of self-report subjective cognitive decline may be limited by lack of awareness about one’s own cognitive abilities in adults with MCI and dementia, and the extent to which discordance between self- and study partner–report is associated with diagnosis of cognitive impairment is unknown.

**Objective:**

To investigate the association between discordance between self- and study partner–reported cognitive and/or functional decline and MCI diagnosis.

**Design, Setting, and Participants:**

This multisite, cross-sectional study used baseline data from 2 longitudinal, observational studies. A total of 921 participant–study partner dyads enrolled in the Alzheimer Disease Neuroimaging Initiative (ADNI) from December 2016 to July 2022, and 279 dyads enrolled in the Brain Health Registry Electronic Validation of Online Methods Study (eVAL) from January 2020 to July 2023 were included.

**Exposures:**

Participants and study partners completed the Everyday Cognition Scale (ECog). Participants completed a demographics survey and the Geriatric Depression Scale-Short Form (GDS).

**Main Outcomes and Measures:**

The model selection procedure in ADNI identified variables, which were included in a model that was externally validated in the eVAL cohort. The primary outcome was MCI vs cognitively unimpaired (CU) among participants.

**Results:**

ADNI participants (921 dyads) had a mean (SD) age of 71 (7) years and mean (SD) of 17 (3) years of education; 485 (53%) were female, 30 (3%) were Asian, 105 (11%) were Black, and 756 (82%) were White. eVAL participants (279 dyads) had a mean (SD) age of 71 (8) years and mean (SD) of 17 (2) years of education; 151 (54%) were female, 17 (6%) were Asian, 12 (4%) were Black, and 245 (88%) were White. The model distinguished CU vs MCI in the validation cohort with an area under the curve of 0.87 (95% CI, 0.88-0.96), sensitivity of 0.50 (95% CI, 0.49-0.80), and specificity of 0.97 (95% CI, 0.95-0.99) based on a regression model. The model included 4 discordance metrics, participant demographics (gender, age, and education), study partner demographics (gender and cohabitation), and depressive symptoms (GDS score).

**Conclusions and Relevance:**

In this cross-sectional study of 1200 dyads, measures of ECog score discordance helped distinguish CU from MCI individuals with high specificity. Participant and study partner agreement on lack of observed changes in the participant was associated with lower likelihood of MCI, highlighting the value of dyadic discordance metrics for ruling out MCI in diverse settings.

## Key Points

**Question:**

Is discordance (disagreement) between self- and study partner–report Everyday Cognition (ECog) scale scores associated with mild cognitive impairment (MCI) diagnosis?

**Findings:**

In this cross-sectional study of 1200 participant–study partner dyads, 4 discordance metrics were independently associated with MCI diagnosis in the model development cohort. Model selection identified variables that distinguished MCI and non-MCI groups in the validation cohort with high accuracy and specificity.

**Meaning:**

These findings suggest that including measures of ECog score discordance may help distinguish cognitively unimpaired individuals from MCI individuals with high specificity.

## Teaser

This cross-sectional study examines whether there is an association of discordance between self- and study partner-reported cognitive and functional decline with mild cognitive impairment diagnosis.

## Introduction

Scalable, efficient tools to identify individuals with likely cognitive impairment is an unmet need in the Alzheimer disease and related dementias field. Subjective report of cognitive and functional decline from participant–study partner (SP) dyads can provide insight into cognitive impairment and clinical progression.^1,2,3,4,5^ SP report has been shown to differentiate between and predict progression from cognitively unimpaired (CU) status to mild cognitive impairment (MCI).^6,7^ Adults with MCI may lack awareness about their cognitive status, causing them to underreport changes.^8,9,10^ Therefore, SP report has been found to be more accurate than self-report in cognitively impaired adults.^3,11^ However, the extent to which disagreement between self- and SP report is associated with MCI diagnosis is not fully understood. In MCI, there is variability in the participant’s awareness of cognitive changes, and the SP may have a more accurate view of the person’s objective cognitive status.^12,13,14,15^ Studies^9,16,17^ have found that participants’ lack of insight into cognitive problems relative to an informant may reflect greater risk of cognitive decline.

Dyadic response data are well-studied in the field of psychometrics.^18^ In this study, we aimed to address whether discordance (disagreement) between self- and SP-reported subjective decline could help identify adults with MCI in in-clinic and remote online settings. We evaluated 4 metrics of dyadic discordance in the Everyday Cognition Scale (ECog),^9,19,20^ an instrument measuring self- and SP-reported cognitive and/or functional decline. These measures were designed to (1) capture directionality of the discordance (whether the SP reported more decline than the participant or vice versa; (2) capture magnitude of discordance; and to further elucidate the role of awareness in MCI, measure composite ECog items in which the participant (3) overreported vs (4) underreported relative to the SP.^9^

We carried out our study in 2 phases, first investigating associations between discordance metrics and diagnostic status in Alzheimer Disease Neuroimaging Initiative (ADNI) participants. We then conducted variable selection to determine the set of features that best distinguished CU from MCI participants. We included the selected variables in a model that was externally validated in a separate cohort, the Brain Health Registry Electronic Validation of Online Methods Study (eVAL) cohort from the UCSF Brain Health Registry (BHR), where the ECog is collected in a remote, digital setting and participants are clinically evaluated for MCI.

## Methods

### Study Samples

This article adheres to the Strengthening the Reporting of Observational Studies in Epidemiology (STROBE) reporting guideline. Our study sample consisted of 2 cohorts: a model selection cohort (ADNI), and a model validation cohort (eVAL). ADNI ran from 2016 to 2022, had approval from each clinical site’s institutional review board (IRB), and all participants signed an informed consent form. eVAL study activities were performed under local and University of California, San Francisco IRB approval. Informed consent was obtained during participants’ in-clinic visit, and via an online consent within the BHR study portal.

#### ADNI

For model selection, we included 921 ADNI3 dyads (eMethods 1 in Supplement 1 describes ADNI). Data were retrieved from the ADNI database at the University of Southern California Laboratory of NeuroImaging.^21^ Inclusion criteria were: (1) had available SP demographic and relationship data, (2) either CU or MCI during initial ADNI3 visit, and (3) had both SP and self-report ECog scores from within 180 days of their first ADNI3 visit. In cases where participants had information for multiple SPs, the SP closest to baseline was used.

#### eVAL

For external model validation, we included 279 dyads from the BHR eVAL study^22,23^ (eMethods 2 in Supplement 1 summarizes eVAL). eVAL participants had both SP and self-report ECog scores from within 180 days of their baseline visit.

### Measures

#### Participant and SP Characteristics

In ADNI and eVAL, the following self-reported demographic information was included: participant and SP age (continuous), participant and SP gender (female or male), participant years of education (continuous), participant ethnicity (Latino or not Latino), participant race (Asian, Black, White, or other). Data on race and ethnicity were collected to evaluate ethnocultural diversity within the sample to better understand the contribution of these factors to self and informant-report of cognitive and functional decline. eMethods 3 in Supplement 1 includes information about collection of race and ethnicity in the studies. For this analysis, we created an other category due to the small number of participants in some of the race categories. The other category included American Indian or Alaskan Native, Hawaiian or Other Pacific Islander, more than 1 race, and unknown or declined to state. Dyad relationship variables included relationship type (spouse, adult child, friend or companion, other relative, and other) and SP living with the participant (yes or no).

#### Geriatric Depression Scale

In ADNI and eVAL, the Geriatric Depression Scale (GDS) Short Form total score was used, which is a self-rated 15-item screening tool used to evaluate depressive symptom severity in older adults.^24,25^ Higher total scores indicate greater depressive symptoms. We excluded the following question from GDS score calculation: “Do you feel you have more problems with memory than most?” since this question may be confounded with report of cognitive decline. The GDS score closest to (obtained within 4 weeks of) baseline ECog assessments was used. eVAL included a digital version identical to the paper version.

#### Everyday Cognition Scale Discordance Metrics

The ECog measures subjective change in cognition and instrumental activities of daily living compared with 10 years before^26^ (eMethods 4 in Supplement 1 includes information about ECog). We derived 4 composite metrics of discordance between participant and SP ECog scores (dyadic discordance): raw ECog score difference (study partner − participant ECog score), absolute ECog score difference (the absolute value of study partner − participant ECog score), overreport Score, and underreport Score (Table 1). Overreport and underreport scores are item-based measures of heightened or lessened awareness of decline in memory and functioning.^9^ Underreport scores (participant lack of awareness) were computed by subtracting the study partner scores from the participant scores for each of the 39 ECog items, then capping each positive item difference at 0 and calculating the mean. We employed the same process for overreport scores (heightened participant awareness), by capping each negative item difference at 0 and taking the mean.

**Table 1.** Everyday Cognition Scale Discordance Metrics Definitions and Examples

Discordance metric	Definition/example	Score output	Considers magnitude of discordance	Considers direction of discordance^a^
Raw difference	The difference between SP and participant ECog scores, eg: Participant score = 2 Study partner score = 1 Raw difference = 1−2 = −1	Continuous values between 3 and −3. Positive scores indicate more decline reported by study partner. Negative scores indicate more decline reported by the participant.	Yes, using average score of all ECog items.	Yes
Absolute difference	The absolute value (magnitude) of the difference between SP and participant ECog scores, eg: Participant score = 2 Study partner score = 1 Raw difference = |1−2| = 1	Continuous values between 1 and 3	Yes, using average score of all ECog items.	No
Overreport score	Calculated based on items in which participants rated themselves as having more decline than their SPs rated them, indicating heightened awareness	Continuous values between −4 and 0	Yes	Yes
Underreport score	Calculated based on items in which the participant rated themselves as having less decline than SPs rated them, indicating less awareness.	Continuous values from 0 to 4	Yes	Yes

^a^ Considers whether the participant reports more decline than the study partner or vice versa.

#### Clinical Diagnosis

In eVAL, the Uniform Data Set, version 3 (UDS)^27^ was administered to all participants. Clinical diagnosis of CU, MCI, or mild dementia was obtained from UDS section D1.

In ADNI, participants undergo diagnostic screening upon enrollment, and a diagnostic category is assigned based on criteria in the ADNI protocol,^28^ which include a global score on the Clinical Dementia Rating assessment.^29^ At subsequent time points, site clinicians judge diagnostic status.

ADNI participants in this analysis come from the ADNI3 phase, including both new participants and those who rolled over from previous phases. The diagnostic status was associated with the first ADNI3 clinical visit in which both SP and self-report ECog scores were collected was used.

### Statistical Analysis

We produced descriptive statistics summarizing and comparing the ADNI and eVAL cohorts. In the ADNI cohort, we fit a logistic regression model for each of the dyadic discordance metrics to independently evaluate their association with MCI diagnosis after adjusting for dyad relationship and sociodemographic factors. Additionally, a Nagelkerke pseudo-*R*^2^ value^30^ was computed for a model consisting exclusively of SP ECog and demographic covariates.

We used a bayesian spike and slab regression model^31^ for variable selection in ADNI. A total of 14 variables collected in both ADNI and eVAL were considered for inclusion: the 4 discordance metrics, participant age, ethnicity, race, gender, education, and GDS score; SP age, gender, and dyad relationship type; and whether the participant and SP lived together, along with all possible 2-way interactions between these terms.

To accommodate the complexity of relationships among variables, the model was structured as a generalized additive model with flexibility to include smoothing splines for some covariates.^32^ Smooth terms in the final selected model were represented with restricted cubic splines.

Variable inclusion was determined based on posterior inclusion probability. Terms with an inclusion probability greater than 0.25 were included in the subsequent validation step. A logistic regression model containing the selected variables was then fit to the eVAL cohort, and area under the curve (AUC) and sensitivity and specificity were calculated to quantify the performance of the model in distinguishing CU vs MCI participants. eMethods 5 in Supplement 1 includes a discussion of the model selection and interpretation. Statistical tests were 2-sided with an α less than .05 considered significant. Welch *t *tests were conducted for continuous variables and Fisher exact tests for categorical variables. Analyses were run in R version 4.3.3 (R Project for Statistical Computing).

## Results

### Participants

**Figure 1.**
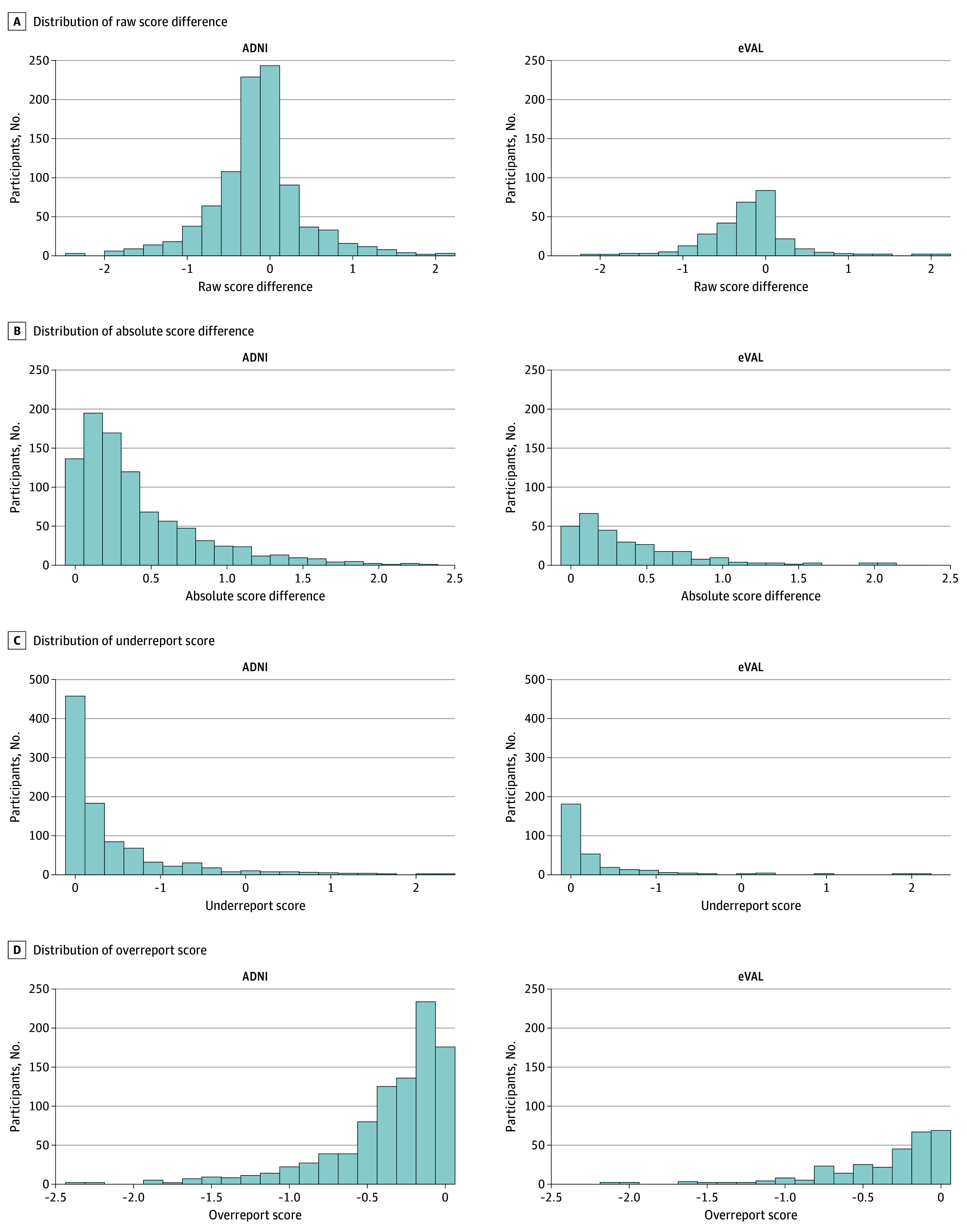
Histograms Displaying Distributions of the 4 Dyadic Discordance Metrics ADNI indicates Alzheimer Disease Neuroimaging Initiative; eVAL, Brain Health Registry Electronic Validation of Online Methods Study.

A total of 921 ADNI dyads had a mean (SD) age of 71 (7) years and a mean (SD) of 17 (3) years of education; 485 (53%) were female, 30 (3%) were Asian, 105 (11%) were Black, and 756 (82%) were White. A total of 573 (62%) were CU and 348 (38%) were MCI. Participants had a mean (SD) self-reported ECog score of 1.57 (0.52) and SP-reported ECog score of 1.42 (0.53) (Figure 1 and Table 2). A total of 279 eVAL dyads had a mean (SD) age of 71 (8) years and a mean (SD) of 17 (2) years of education; 151 (54%) were female, 17 (6%) were Asian, 12 (4%) were Black, and 245 (88%) were White. A total of 225 (81%) were CU and 54 (19%) were MCI. Participants had a mean (SD) self-reported ECog score of 1.44 (0.45) and SP-reported ECog score of 1.22 (0.37) (Table 2).

**Table 2.** Characteristics of Dyads Enrolled in the Alzheimer Disease Neuroimaging Initiative (ADNI) and the Brain Health Registry Electronic Validation of Online Methods Study (eVAL) Study

Characteristic	Dyads, No. (%)		*P* value^a^
	ADNI (n = 921)	eVAL (n = 279)	
Raw ECog score difference, mean (SD)	−0.16 (0.54)	−0.22 (0.48)	.07
Absolute ECog score difference, mean (SD)	0.40 (0.40)	0.36 (0.38)	.10
Participant ECog overreport score, mean (SD)	−0.35 (0.36)	−0.32 (0.35)	.08
Participant ECog underreport score, mean (SD)	0.19 (0.30)	0.12 (0.25)	<.001
Participant ECog score, mean (SD)	1.57 (0.52)	1.44 (0.45)	<.001
Participant characteristics			
Participant diagnostic category			
CU	573 (62)	225 (81)	<.001
MCI	348 (38)	54 (19)	
Participant baseline age, y, mean (SD)	71 (7)	71 (8)	.05
Participant ethnicity			
Latino	68 (7)	10 (4)	.03
Not Latino	853 (93)	267 (96)	
Missing	0	2 (<1)	
Participant Geriatric Depression Scale score, mean (SD)	1.32 (1.71)	1.53 (2.13)	.70
Participant gender			
Female	485 (53)	151 (54)	.70
Male	436 (47)	128 (46)	
Participant race			
Asian	30 (3)	17 (6)	<.001
Black	105 (11)	12 (4)	
White	756 (82)	245 (88)	
Other^b^	30 (3)	4 (1)	
Missing	0	2 (<1)	
Participant years of education, mean (SD)	17 (3)	17 (2)	.05
Study partner characteristics			
Study partner baseline age, mean (SD)	67 (12)	67 (11)	.90
Study partner ECog score, mean (SD)	1.42 (0.53)	1.22 (0.37)	<.001
Study partner gender			
Female	610 (66)	168 (60)	.08
Male	311 (34)	110 (40)	
Missing	0	1 (<1)	
Dyad relationship characteristics			
Study partner lives with participant	640 (70)	190 (68)	.70
Study partner relationship			
Spouse	570 (62)	178 (64)	.80
Other	21 (2.3)	7 (2.5)	
Other relative	65 (7.1)	21 (7.6)	
Adult child	136 (15)	33 (12)	
Friend/companion	129 (14)	39 (14)	
Missing	0	1 (<.01)	

Abbreviations: CU, cognitively unimpaired; ECog, Everyday Cognition; MCI, mild cognitive impairment.

^a^ Two-sample *t* tests for continuous variables; Pearson χ^2^ tests for categorical variables.

^b^ Other included American Indian or Alaskan Native, Hawaiian or Other Pacific Islander, more than 1 race, and unknown or declined to state.

### Association Between Discordance Metrics and MCI Status in the ADNI Cohort

In the ADNI cohort, greater odds of MCI diagnosis were associated with higher raw ECog score difference (standardized odds ratio (sOR), 1.28; 95% CI, 1.10-1.48), higher absolute ECog score difference (sOR, 1.88; 95% CI, 1.60-2.23), higher participant ECog underreport score (sOR, 2.64; 95% CI, 2.14-3.32), and lower participant ECog overreport score (sOR, 0.76; 95% CI, 0.65-0.89). Greater odds of MCI diagnosis were also associated with higher self- and SP-reported ECog scores (self: sOR, 2.05; 95% CI, 1.65-2.56; SP: sOR, 3.98; 95% CI, 3.06-5.28). See Table 3 for ORs and directionality of the discordance metrics in the included covariates. A Nagelkerke pseudo-*R*^2^ value of 0.43 was computed for a model consisting of SP ECog and demographic covariates, and the addition of ECog score difference to that model resulted in a value of 0.48, suggesting the addition of discordance to the model resulted in a greater proportion of explained variance in diagnostic status.

**Table 3.** Estimated Odds Ratios (ORs) and 95% CIs From Logistic Regression Models Fit to Participant Mild Cognitive Impairment Diagnosis in the Alzheimer Disease Neuroimaging Initiative Cohort

Model and dependent variable^a^	Standardized OR (95% CI)
Model with ECog score difference and covariates	
ECog score difference	1.28 (1.10-1.48)^b^
Participant female gender	0.80 (0.65-0.97)^b^
Participant age	1.28 (1.10-1.50)^b^
Participant years of education	0.73 (0.65-0.84)^b^
Participant GDS score	2.10 (1.76-2.51)^b^
Participant race	
Asian	0.46 (0.20-0.96)^b^
Black	1.09 (0.67-1.79)
White	1 [Reference]
Other^c^	1.38 (0.66-2.83)
Participant Latino ethnicity	0.90 (0.64-1.24)
Study partner female gender	1.12 (0.91-1.38)
Participant lives together with study partner	1.23 (1.02-1.49)^b^
Model with absolute ECog score difference plus covariates	
Absolute ECog score difference	1.88 (1.60-2.23)^b^
Participant female gender	0.80 (0.65-0.98)^b^
Participant age	1.27 (1.08-1.49)^b^
Participant years of education	0.74 (0.63-0.86)^b^
Participant GDS score	1.76 (1.49-2.09)^b^
Participant race	
Asian	0.44 (0.18-0.93)^b^
Black	1.31 (0.79-2.22)
White	1 [Reference]
Other^c^	1.07 (0.48-2.32)
Participant Latino ethnicity	1.09 (0.77-1.52)
Study partner female gender	1.17 (0.95-1.44)
Participant lives together with study partner	1.31 (1.08-1.60)^b^
Model with participant ECog underreport score plus covariates	
Participant ECog underreport score	2.64 (2.14-3.32)^b^
Participant female gender	0.80 (0.64-0.98)^b^
Participant age	1.26 (1.07-1.48)^b^
Participant years of education	0.76 (0.65-0.89)^b^
Participant GDS score	1.93 (1.62-2.32)^b^
Participant race	
Asian	0.51 (0.21-1.09)
Black	1.21 (0.73-2.03)
White	1 [Reference]
Other^c^	1.23 (0.56-2.60)
Participant Latino ethnicity	0.92 (0.65-1.29)
Study partner female gender	1.06 (0.85-1.31)
Participant lives together with study partner	1.14 (0.94-1.40)
Model with participant ECog overreport score plus covariates^d^	
Participant ECog overreport score	0.76 (0.65-0.89)^b^
Participant female gender	0.79 (0.65-0.97)^b^
Participant age	1.28 (1.09-1.49)^b^
Participant years of education	0.72 (0.62-0.84)^b^
Participant GDS score	1.84 (1.55-2.20)^b^
Participant race	
Asian	0.46 (0.20-0.95)^b^
Black	1.16 (0.71-1.91)
White	1 [Reference]
Other^c^	1.23 (0.58-2.59)
Participant Latino ethnicity	1.00 (0.71-1.37)
Study partner female gender	1.17 (0.95-1.44)
Participant lives together with study partner	1.29 (1.07-1.56)^b^
Model with participant ECog plus study partner ECog score plus covariates	
Participant ECog score	2.05 (1.65-2.56)^b^
Study partner ECog score	3.98 (3.06-5.28)^b^
Participant female gender	0.74 (0.59-0.94)^b^
Participant age	1.27 (1.06-1.52)^b^
Participant years of education	0.80 (0.66-0.95)^b^
Participant GDS score	1.21 (0.99-1.47)
Participant race	
Asian	0.42 (0.15-1.06)
Black	1.95 (1.11-3.51)^b^
White	1 [Reference]
Other^c^	1.01 (0.41-2.38)
Participant Latino ethnicity	1.08 (0.73-1.57)
Study partner female gender	1.01 (0.80-1.28)
Participant lives together with study partner	1.05 (0.85-1.31)

Abbreviations: ECog, Everyday Cognition; GDS, Geriatric Depression Scale.

^a^ Each model contains a single discordance metric and is adjusted for participant baseline age, participant race, participant gender, study partner gender, participant education level, participant Geriatric Depression Score, and whether study partner lives with participant.

^b^ *P* < .05.

^c^ Other included American Indian or Alaskan Native, Hawaiian or Other Pacific Islander, more than 1 race, and unknown or declined to state.

^d^ Participant ECog overreport score is negative, with a lower overreport score indicating a greater degree of overreporting.

### Model Selection in ADNI Cohort

Using a regression model, we selected a final model that included all 4 discordance metrics, participant demographics (gender, age, and educational attainment), SP gender, whether the SP and participant lived together, and GDS score. The variable selection method indicated a nonlinear effect of raw and ECog score difference.

### External Model Validation in the eVAL Cohort

**Figure 2.**
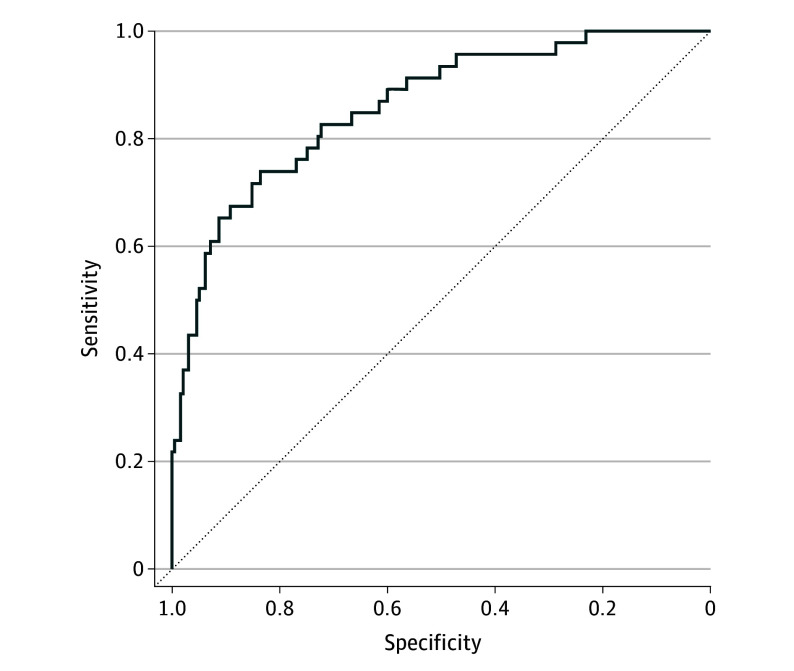
Receiver Operating Characteristic Curve for Diagnostic Classification Performance of the Alzheimer Disease Neuroimaging Initiative Selected Model in the Brain Health Registry Electronic Validation of Online Methods Cohort

This model distinguished CU vs MCI groups in the eVAL cohort of 279 dyads with an AUC of 0.87 (95% CI, 0.88-0.96), sensitivity of 0.50 (95% CI, 0.49-0.80), and specificity of 0.97 (95% CI, 0.95-0.99) (Figure 2). A model consisting only of demographic variables and other participant characteristics resulted in an AUC of 0.65, compared with a minimum AUC of 0.74 for models that contained a discordance variable.

## Discussion

Major findings were (1) significant associations between diagnostic status (CU vs MCI) and multiple measures of dyadic discordance, defined as difference between self- and SP-report of cognitive and functional decline; (2) that a combination of discordance variables, participant demographics, depressive symptoms, and dyad relationship variables best distinguished CU vs MCI participants in the ADNI cohort; and (3) that external validation of the selected model in a remotely assessed cohort with clinically confirmed diagnoses distinguished diagnostic groups with moderate accuracy, supporting the generalizability of the approach. These results suggest participant and study partner agreement on lack of observed changes in the participant is associated with lower likelihood of MCI and that dyadic subjective cognitive decline (SCD) discordance can help rule out MCI in in-clinic and remote settings.

The first finding was that multiple dyadic discordance measures were associated with MCI diagnosis. Many studies have considered only self-report,^33,34^ or a combination of self and SP report.^2,35^ Few have investigated the role of discordance, although the dynamic relationship between self and SP report over the clinical continuum from CU to dementia suggests that it may be important.

Discordance and mutual report metrics were significantly associated with MCI diagnosis. Higher raw ECog score difference, higher absolute value of ECog score difference, and both greater participant underreporting and overreporting of decline were all associated with greater odds of MCI diagnosis. These findings suggest that multiple discordance measures provide useful information about an independent underlying construct related to dyadic subjective cognitive decline.

We investigated multiple discordance measures because there is no consensus on the most clinically meaningful measure. The raw difference metric considers the magnitude and direction of discordance, with positive values indicating that the SP reported more overall decline than the participant, and negative values indicating that the participant reported more decline than the SP. The absolute value of the difference considers the magnitude of discordance, but not the direction. Difference scores for ECog have previously been shown to illustrate longitudinal change in participants’ memory awareness. In a separate study,^20^ difference scores (informant minus participant score on the Daily Function Questionnaire) were higher in individuals with dementia compared with MCI and CU individuals. However, the study showed no significant difference between MCI and CU scores. Greater unawareness, or underreport scores, were shown to be associated with risk of clinical progression in a cohort of cognitively normal ADNI participants.^9^ In terms of combined self- and SP-(mutual) report, research has demonstrated a relationship between self and informant report and risk of incident dementia.^36^

We also identified dyadic sociodemographic and relationship variables associated with the ability of discordance metrics to classify CU vs MCI. Associations with participant demographics to SCD are understudied, although recent evidence suggests differences in SCD in minoritized ethnocultural groups, including a weaker relationship between SCD and objective measures of cognition in some groups.^37,38,39,40,41,42,43,44^ For participant demographics, for some discordance metrics, advanced age, male gender, and lower education were all significantly associated with greater odds of MCI diagnosis. These associations varied across the discordance metrics (Table 3). Previous research has shown higher prevalence of SCD in older adults and individuals with female gender.^45^ Another study found that Black and Hispanic individuals with SCD were more likely to be younger, have lower educational attainment, and have lower income.^46,47,48^ However, associations of race, ethnicity, and other sociodemographic factors with subjective report are not well understood.

Few studies consider SP characteristics when investigating SCD and their relationship to AD-related outcomes. Although we found no association with SP ethnocultural identity, the lack of ethnocultural diversity in our cohorts limits our ability to investigate possible associations. In fact, others have found that informant report can vary across different ethnocultural groups, potentially in part due to differing perceptions of and cultural attitudes toward dementia, and differences in how groups endorse concerns pertaining to cognition.^49,50,51,52^

Regarding dyad relationship, we found higher odds of MCI diagnosis in dyads who live together vs those who do not. This suggests that greater dyad familiarity contributes to the ability of discordance metrics to identify those with MCI, as lack of familiarity is a potential source of measurement error in the study partner ECog. The finding is consistent with a past finding from BHR showing that the relationship between SP ECog and self-report diagnosis is stronger in dyads who cohabitate.^6^ Unlike past studies, we did not find an association between relationship type (spouse or other).^53,54^ Thus, cohabitation may be a more important relationship metric than relationship type when using SCD to distinguish CU from MCI.

Finally, in line with our findings, studies have suggested that depressive symptoms are both a risk factor for cognitive and functional decline^55^ and also a consequence of neurodegenerative disease and/or MCI.^56^ As such, as our results suggest using measures of depressive symptoms that do not require a clinician for administration offer advantages for identifying individuals with MCI in remote assessments, particularly when combined with discordance measures.

Using ADNI data, we identified variables (discordance, demographics, and relationship) that best distinguished CU from MCI participants. When we applied this model to a separate cohort with a lower prevalence of MCI (38% in model selection cohort vs 19% in the validation cohort), the results were similar, supporting the external validity of our approach. The model exhibited high specificity (95%) but low sensitivity (50%). This is likely due to sample characteristics, including a high proportion of CU participants in the eVAL cohort.

The choice of variable selection and validation as compared with fitting a mixed-effects model with a cohort-level random effect serves several purposes. First, it reduces the risk of overfitting by confirming that the findings are not merely artifacts of the much larger ADNI cohort. Second, it helps to confirm the robustness and relevance of the selected variables in a separate sample. Finally, it better elucidates how well these metrics generalize from an in-clinic to a remote setting.

Post hoc comparison of false negatives and true positives in eVAL sheds light on the low sensitivity. The false negatives (MCI participants who the model did not classify as impaired) had significantly lower average SP ECog scores compared with true positives (*P* < .05), despite not including SP ECog in the analysis. True positives were significantly more likely to have spousal SPs and female SPs. Participant ECog scores were not significantly different between the cohorts. These findings suggest 2 sources of measurement error in the participant and SP versions of the instrument: unawareness within the participant ECog, and lack of familiarity with the participant within the SP ECog.

### Limitations

Multiple selection biases limit the generalizability of our findings, including lack of ethnocultural and/or educational diversity in both cohorts and the requirement for access to an internet-connected device and digital literacy in the eVAL cohort. ADNI and eVAL are samples of convenience with additional selection biases. Requiring an SP is a bias in both samples, and likely disproportionately affects those from underincluded ethnocultural groups.^57^ In ADNI, SP demographic and relationship information was only available in ADNI3, which may bias our sample toward those healthy enough to roll over into ADNI3 from previous study phases.

The low sensitivity is a limitation when considering use of discordance metrics as a screening measure. Low sensitivity is attributable in part to lack of dyad familiarity; sensitivity was higher in dyads who cohabitated. In clinical trials there are often stricter requirements for familiarity. Future studies are needed to see if the discordance metrics could be useful in settings like trials. Conversely, the high specificity could be useful for ruling out cognitive impairment in various settings.

## Conclusions

In this cross-sectional study, our results identified measures of discordance between self- and SP-report subjective cognitive and functional decline that may be useful for detecting true negatives, that is, individuals who do not have MCI in remote or digital and in-clinic settings, dementia studies, trials, and health care environments. Future longitudinal studies could investigate whether changes in discordance metrics are correlated with disease progression.

## Notes

Supplement 1. **eMethods 1. **Brief Description of This Alzheimer’s Disease Neuroimaging Initiative **eMethods 2.** Description of Electronic Validation of Online Methods to Predict and Monitor Cognitive Decline study **eMethods 3.** Detailed Description of the Collection of Race and Ethnicity Data **eMethods 4.** Additional information About the Everyday Cognition Scale **eMethods 5.** Statistical Methods

Supplement 2.
Data Sharing Statement
