# Supplementary material for: Self- and Informant-Report Cognitive Decline Discordance and Mild Cognitive Impairment Diagnosis
Source: JAMA Netw Open. 2025 Apr 18;8(4):e255810. doi: 10.1001/jamanetworkopen.2025.5810 (PMC12008764; doi:10.1001/jamanetworkopen.2025.5810)
Supplement: Supplement 1. — eMethods 1. Brief Description of This Alzheimer’s Disease Neuroimaging Initiative eMethods 2. Description of Electronic Validation of Online Methods to Predict and Monitor Cognitive Decline study eMethods 3. Detailed Description of the Collection of Race and Ethnicity Data eMethods 4. Additional information About the Everyday Cognition Scale eMethods 5. Statistical Methods [file jamanetwopen-e255810-s001.pdf]

Supplemental Online Content

Aaronson A, Diaz A, Ashford MT, et al. Self- and informant-report cognitive decline discordance with mild cognitive impairment diagnosis. *JAMA Netw Open*. 2025;8(4):e255810. doi:10.1001/jamanetworkopen.2025.5810

eMethods 1. Brief Description of This Alzheimer's Disease Neuroimaging Initiative

eMethods 2. Description of Electronic Validation of Online Methods to Predict and Monitor Cognitive Decline study

eMethods 3. Detailed Description of the Collection of Race and Ethnicity Data

eMethods 4. Additional information About the Everyday Cognition Scale

eMethods 5. Statistical Methods

This supplemental material has been provided by the authors to give readers additional information about their work.

© 2025 Aaronson A et al. *JAMA Network Open*.

eMethods 1. Brief Description of this Alzheimer's Disease Neuroimaging Initiative

Launched in 2003, the Alzheimer's Disease Neuroimaging Initiative (ADNI) is a public private partnership, led by the Principal Investigator Michael W. Weiner, MD and is an ongoing, multicenter, longitudinal study with the overall aim of developing and validating imaging, genetic, biochemical, and clinical biomarkers for Alzheimer's disease clinical trials[1–4]. Participants enrolled in ADNI need to be aged 55 to 90 and are either classified as cognitively unimpaired, having mild cognitive impairment or dementia due to Alzheimer's disease. So far, there have been five phases (ADNI1, ADNI-GO, ADNI2, ADNI3, ADNI4). For up-to-date information, see www.adni-info.org.

© 2025 Aaronson A et al. *JAMA Network Open*.

eMethods 2. Description of Electronic Validation of Online Methods to Predict and Monitor Cognitive Decline study

The Brain Health Registry (BHR) Electronic Validation of Online Methods to Predict and Monitor Cognitive Decline (eVal) study ran from 2020-2023. eVAL Participants were recruited from two sources: (1) The University of California, San Francisco Brain (UCSF) Health Registry (BHR)[5,6]; and (2) National Institute on Aging Alzheimer's Disease Research Centers (ADRCs) at Washington University in St. Louis, the Mayo Clinic in Rochester Minnesota, and University of Alabama at Birmingham. All participants were age 55 and older and fluent in English. Participants were required to have a study partner and be proficient using an Internet-connected device, such as a smartphone or computer. Exclusion criteria, assessed during screening, were self-report of an acute or uncontrolled major medical condition, and recent history (< 6 months) of abuse or dependence on drugs and/or alcohol. All study partners were age 18 or older, fluent in English and access to and proficiency using an Internet-connected device. Study partners were required to have regular and frequent interaction (either online, by telephone, or in person) with the participant, such that they could answer questions about the participant's memory and day-to-day functioning. At UCSF, current BHR participants were referred to the study via a series of four automated email invitations with the additional inclusion criteria: (1) Agreed to be emailed about opportunities to participate in additional research; (2) Located within 50 miles of the clinic site. The three other sites recruited ADRC participants during regular clinical visits.

© 2025 Aaronson A et al. *JAMA Network Open*.

eMethods 3. Detailed description of the collection of race and ethnicity data

In ADNI, participant race is self-reported using the following categories: American Indian/Alaskan Native, Asian, Black, Hawaiian/Other Pacific Islander, More than one, Unknown, White. Self-reported ethnicity was collected using the following two categories: Hispanic/Latino, Not Hispanic/Latino). In BHR, race is self-reported with the following options: African American, Asian, Caucasian, Native American, Pacific Islander, Other, Declined to State) and ethnicity is self-reported using the following categories: Latino, Not Latino, Declined to state).

© 2025 Aaronson A et al. *JAMA Network Open*.

eMethods 4. Additional information about the Everyday Cognition Scale

The Everyday Cognition Scale (eCog)[7] is completed separately by the participant and study partner, and includes questions related to six cognitive domains: Everyday Memory (i.e., remembering where objects have been placed), Everyday Language (i.e., change in verbally giving instructions to others), Everyday Visuospatial Abilities (i.e., finding the way around a familiar store), Everyday Planning (i.e., developing a schedule in advance of anticipated events), Everyday Organization (i.e., change in prioritizing tasks by importance), and Everyday Divided Attention (i.e., change in returning to a task after being interrupted). ECog scores range from 1-4, with higher scores indicating greater decline. BHR uses a version adapted for online use[8]. This analysis used baseline ECog scores from ADNI and BHR

© 2025 Aaronson A et al. *JAMA Network Open*.

eMethods 5. Statistical Methods

In this supplement we dedicate more space to expanding on the statistical methodology used in the study, explore the results of the model selection procedure in greater detail, and examine post-hoc analyses to better interpret the findings.

1. Model Selection

The problem of selecting the “best” subset of covariates to optimize the performance of a statistical model is a long-studied problem that exists under many names, including variable selection, subset selection, feature selection, and model selection. Numerous methodologies for addressing this problem have been developed and studied[9].

A very common framework for approaching the selection problem is regularized regression - modification of the traditional OLS objective function by including an additive penalty term to discourage overfitting and shrink estimated regression coefficients to zero.

Well known examples of this procedure include l1-penalized regression, better known as the LASSO, l2-penalized ridge regression, and the more general elastic net regression framework, in which the penalty term is a weighted average of l1 and l2 penalties[10,11]. These frameworks have been extensively studied in both the theoretical and applied statistics literature for the better part of a half century, and its efficacy is well-established, particularly in cases where the solution set is extremely sparse in the parameter space and there is little dependence between candidate variables[12].

© 2025 Aaronson A et al. *JAMA Network Open*.

Another method of model selection that readers may be familiar with is that of stepwise selection, in which a list of variables is fit iteratively, in either a backwards or forwards manner, and the procedure continues until it is halted according to some selection criteria - commonly utilized metrics include the Akaine information criteria or the closely related Bayesian information criteria, and stopping rules based on significance testing[13]. Despite its enduring popularity in the medical literature, this method is widely considered to be statistically unsound[14]. Formally, stepwise selection can be viewed as an approximation to best subset (BSS) selection, which is itself a form of penalized regression involving an l0 pseudonorm penalty[15]. BSS itself is another method of variable selection; however, its application is limited by computational concerns – as the search space of models scales exponentially in the number of covariates.

Another approach to the problem of variable selection comes from the statistical learning literature. These are generally nonparametric methods including random forests, support vector machines, and gradient-boosted regression. In applications to variable selection, terms for inclusion can be decided based on a number of metrics, including importance functions and kernel estimators[16].

2. Bayesian spike and slab regression

The method we chose for this study is a Bayesian approach to the model selection problem. The model itself is a standard Bayesian regression model, with a particular hierarchical prior setup that induces a sparse set of covariates[17].

© 2025 Aaronson A et al. *JAMA Network Open*.

This is a very general framework that shares some advantages of the previously described methods. The hierarchical prior setup allows us to consider both linear predictors and nonlinear transformations of predictors, including P-splines and multiplicative interactions between terms. We are working with a relatively small set of predictors, many of which are highly correlated, a situation for which LASSO is ill-suited. In addition, we would like to be able to interpret the results of the selection procedure, a requirement for which statistical learning methods are ill suited.

The specific parameterization of spike and slab regression used in the study utilizes a penalized normal mixture of inverse gammas (peNMIG) prior of the form

$$\beta_j | \gamma, \ \tau^2 \ \sim \ N\left(0, \ \tau^2\gamma\right)$$
$$\gamma \mid w \sim w \, \mathcal{I}_1(\gamma) + (1 - w) \, \mathcal{I}_{\nu_0}(\gamma)$$

A specification which is described in full detail here[18]. We selected hyperparameters of the form

$$\tau^2 \sim \Gamma^{-1}(20, 30)$$
$$w \sim \mathrm{Beta}(12, 4)$$

This choice of hyperparameters was made to induce a rather broad spike and centered slab, with the majority of the prior density clustered around 0. A prior density plot is shown below:

© 2025 Aaronson A et al. *JAMA Network Open*.

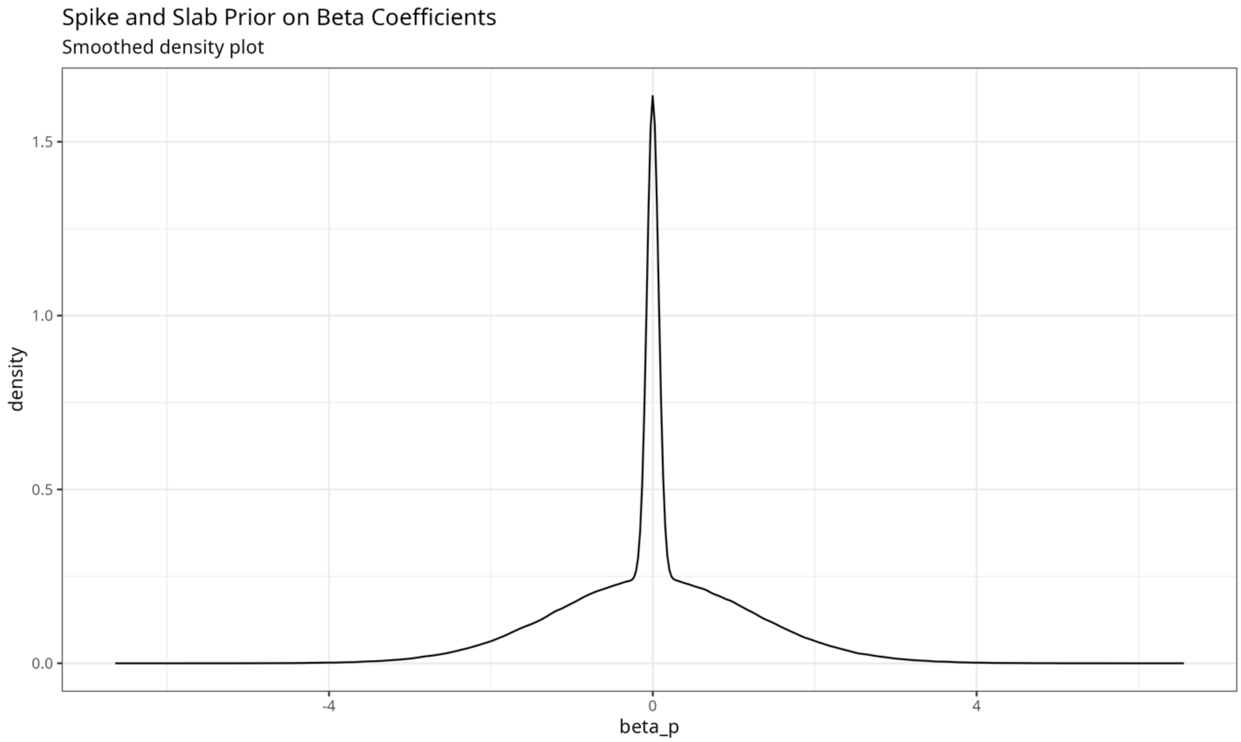

The model was fit to the ADNI cohort data using the R package spikeSlabGAM[19]. This package utilizes a block gibbs sampler, and for the purposes of this study we used 8 chains of length 10,000, and 5000 iterations of burn-in per chain.

The variables included in the selection procedure were underreport score, raw ECog score difference, overreport score, absolute difference in ECog scores, participant GDS score, participant and study partner gender, participant and study partner age, participant education level, study partner relationship and cohabitation status, and participant's status as a self-identified member of a group other than non-Latino White.

In addition to the listed variables as linear predictors, the search space also consisted of all possible two-way interactions between variables, and nonlinear functions (low order P-splines) of the continuous variables. Final variable selection was made on the basis of posterior inclusion probability: the proportion of posterior samples in which a variable

© 2025 Aaronson A et al. *JAMA Network Open*.

was included in the model. For two-way interaction terms, the principle of hierarchy was observed – that is, any model containing an interaction of the form X*Y must also contain X and Y as additive predictors.

The table below summarizes the inclusion probabilities for the predictors that were ultimately selected. Note that, for continuous predictors, sm() indicates that a smoothed version of the predictor was selected while lin() indicates a linear predictor. Factors(denoted 'fct') are not subject to any smoothing, and two-way interactions between X and Y are denoted with X:Y

**Var**	**P(gamma = 1)**
sm(ECog Score Difference):lin(Participant ECog Overreport Score)	0.999
lin(Participant Geriatric Depression Scale Score)	0.979
fct(Participant Gender)	0.957
lin(Participant ECog Underreport Score):sm(Participant Age)	0.819
sm(Participant Age)	0.696
sm(Participant Age):fct(Participant/Study Partner Relationship)	0.655
sm(ECog Score Difference)	0.626

© 2025 Aaronson A et al. *JAMA Network Open*.

lin(Participant Age):fct(Participant lives together with study partner)	0.420
lin(Participant ECog Underreport score)	0.381

For the final fit to the validation cohort, the P-splines used in the variable selection process were replaced with low-order restricted cubic splines for the sake of improved interpretability.

3. Post-hoc evaluation of model performance in validation cohort

The selected model demonstrated high specificity in the validation cohort, but lower sensitivity.

To begin with, the ADNI cohort is enriched for impaired individuals, and as such it represents a less healthy population compared to the BHR eVAL cohort – as a result, we would expect to see very few positive results, whether true or false. In the subset of the eVAL cohort where there was sufficient data to fit the model, there were an equal number of false positives and false negatives, with 23 of each, leading to an overall sensitivity of 0.5.

A post-hoc exploratory analysis was conducted to investigate whether there were any traits that separated the false negatives and true positives. A comparison of summary statistics between the two outcomes is given in the table below. All continuous variables are presented as mean(sd), and all categorical variables are given as n(%).

© 2025 Aaronson A et al. *JAMA Network Open*.

Characteristic	False Negative, N = 23	True Positive, N = 23
Raw ECog Score Difference	-0.34 (0.40)	0.15 (0.97)
Overreport Score	-0.41 (0.35)	-0.36 (0.52)
Underreport Score	0.09 (0.10)	0.52 (0.61)
Absolute ECog Score Difference	0.40 (0.34)	0.75 (0.62)
Participant GDS Score	1.70 (2.14)	1.91 (2.17)
Participant Gender		
Female	13 (57%)	8 (35%)
Male	10 (43%)	15 (65%)
SP Gender		
Female	12 (52%)	21 (91%)
Male	11 (48%)	2 (8.7%)
Participant Age	75 (6)	73 (8)
Participant/SP Cohabitation	14 (61%)	18 (78%)
Participant Years of Education	16.52 (2.41)	17.17 (2.61)

© 2025 Aaronson A et al. *JAMA Network Open*.

Note that this table only includes variables that were included in the selected model. Some other variables which could potentially explain some variation are the participant and study partner ECog scores themselves, as well as the Study partner relationship type, which we have summarized in the tables below:

SP/Participant Relationship	False Negative, N = 23	True Positive, N = 23
Spouse	10 (43%)	17 (74%)
Other	1 (4.3%)	0 (0%)
Other relative	2 (8.7%)	0 (0%)
Adult child	5 (22%)	3 (13%)
Friend/Companion	5 (22%)	3 (13%)

Characteristic	False Negative, N = 23	True Positive, N = 23
Study Partner ECog Score	1.32 (0.27)	1.85 (0.68)

© 2025 Aaronson A et al. *JAMA Network Open*.

Participant ECog Score	1.66 (0.51)	1.71 (0.69)

While it would be inappropriate to conduct any kind of statistical hypothesis test between these two groups due to the inherent selection bias, we can learn from the comparison. A few notable points are presented below:

- Of the participants who were correctly classified as impaired, 91% had study partners who self-identified as female, compared to 52% among false negatives.
- More participants with Spousal SPs were misclassified as unimpaired.
- The Average SP ECog score was lower in the misclassified group compared to the true positives, indicating less perceived decline on the part of the study partner. Correspondingly, the average underreport score among true positives was much higher than among false negatives.

Taking all of these results together, we hypothesize that lack of dyad familiarity is a major source of measurement error in categorizing participants as impaired/unimpaired. Anosognosia on the part of a participant, and lack of attention on the part of the SP, could work together to create situations in which there is little to no signal to be gained.

© 2025 Aaronson A et al. *JAMA Network Open*.

Online-only Supplement References

1. Weiner MW, Veitch DP, Aisen PS, et al. The Alzheimer's Disease Neuroimaging Initiative 3: Continued innovation for clinical trial improvement. *Alzheimers Dement J Alzheimers Assoc*. 2017;13(5):561-571. doi:10.1016/j.jalz.2016.10.006

2. Weiner MW, Aisen PS, Jack CR, et al. The Alzheimer's disease neuroimaging initiative: progress report and future plans. *Alzheimers Dement J Alzheimers Assoc*. 2010;6(3):202-211.e7. doi:10.1016/j.jalz.2010.03.007

3. Weiner MW, Veitch DP, Aisen PS, et al. Impact of the Alzheimer's Disease Neuroimaging Initiative, 2004 to 2014. *Alzheimers Dement J Alzheimers Assoc*. 2015;11(7):865-884. doi:10.1016/j.jalz.2015.04.005

4. Veitch DP, Weiner MW, Aisen PS, et al. Understanding disease progression and improving Alzheimer's disease clinical trials: Recent highlights from the Alzheimer's Disease Neuroimaging Initiative. *Alzheimers Dement J Alzheimers Assoc*. 2019;15(1):106-152. doi:10.1016/j.jalz.2018.08.005

5. Weiner MW, Aaronson A, Eichenbaum J, et al. Brain health registry updates: An online longitudinal neuroscience platform. *Alzheimers Dement J Alzheimers Assoc*. 2023;19(11):4935-4951. doi:10.1002/alz.13077

6. Weiner MW, Nosheny R, Camacho M, et al. The Brain Health Registry: An internet-based platform for recruitment, assessment, and longitudinal monitoring of

© 2025 Aaronson A et al. *JAMA Network Open*.

participants for neuroscience studies. *Alzheimers Dement J Alzheimers Assoc*. 2018;14(8):1063-1076. doi:10.1016/j.jalz.2018.02.021

7. Farias ST, Mungas D, Reed BR, et al. The measurement of everyday cognition (ECog): scale development and psychometric properties. *Neuropsychology*. 2008;22(4):531-544. doi:10.1037/0894-4105.22.4.531

8. Nosheny RL, Camacho MR, Insel PS, et al. Online study partner-reported cognitive decline in the Brain Health Registry. *Alzheimers Dement N Y N*. 2018;4:565-574. doi:10.1016/j.trci.2018.09.008

9. Claeskens G. Statistical Model Choice. *Annu Rev Stat Its Appl*. 2016;3(Volume 3, 2016):233-256. doi:10.1146/annurev-statistics-041715-033413

10. Tibshirani R. Regression Shrinkage and Selection Via the Lasso. *J R Stat Soc Ser B Methodol*. 1996;58(1):267-288. doi:10.1111/j.2517-6161.1996.tb02080.x

11. Zou H, Hastie T. Regularization and Variable Selection Via the Elastic Net. *J R Stat Soc Ser B Stat Methodol*. 2005;67(2):301-320. doi:10.1111/j.1467-9868.2005.00503.x

12. Freijeiro-González L, Febrero-Bande M, González-Manteiga W. A critical review of LASSO and its derivatives for variable selection under dependence among covariates. 2020;(arXiv:2012.11470). doi:10.48550/arXiv.2012.11470

13. Zhang Z. Variable selection with stepwise and best subset approaches. *Ann Transl Med*. 2016;4(7):136. doi:10.21037/atm.2016.03.35

© 2025 Aaronson A et al. *JAMA Network Open*.

14. Smith G. Step away from stepwise. *J Big Data*. 2018;5(1):32. doi:10.1186/s40537-018-0143-6

15. Furnival G, Wilson R. Regressions by leaps and bounds. *Technometrics*. 2000;42:69-79. doi:10.2307/1271435

16. Hastie T, Tibshirani R, Friedman J. *The Elements of Statistical Learning*. Springer; 2009. doi:10.1007/978-0-387-84858-7

17. Ishwaran H, Rao JS. Spike and slab variable selection: Frequentist and Bayesian strategies. *Ann Stat*. 2005;33(2). doi:10.1214/009053604000001147

18. Scheipl F. Normal-Mixture-of-Inverse-Gamma Priors for Bayesian Regularization and Model Selection in Structured Additive Regression Models. doi:10.5282/ubm/epub.11785

19. Scheipl F, Gruen B. spikeSlabGAM: Bayesian Variable Selection and Model Choice for Generalized Additive Mixed Models. Published online October 22, 2024. Accessed January 29, 2025. https://cran.r-project.org/web/packages/spikeSlabGAM/index.html

© 2025 Aaronson A et al. *JAMA Network Open*.
