# Supplementary material for: Self- and Informant-Report Cognitive Decline Discordance and Mild Cognitive Impairment Diagnosis
Source: JAMA Netw Open. 2025 Apr 18;8(4):e255810. doi: 10.1001/jamanetworkopen.2025.5810 (PMC12008764; doi:10.1001/jamanetworkopen.2025.5810)
Supplement: Supplement 2. — Data Sharing Statement [file jamanetwopen-e255810-s002.pdf]

Data Sharing Statement

Aaronson. Self- and Informant-Report Cognitive Decline Discordance With Mild Cognitive Impairment Diagnosis. *JAMA Netw Open*. Published April 18, 2025. doi:10.1001/jamanetworkopen.2025.5810

Data
Data available: Yes
Data types: Deidentified participant data
How to access data: For ADNI participants, we will not be sharing data. Those wishing to access ADNI data must apply for access directly through LONI (https://adni.loni.usc.edu/data-samples/). For eVAL participants, de-identified data can be requested via email (investigators@brainhealthregistry.org). Instructions can be obtained here: https://www.brainhealthregistry.org/for-investigators/de-identified-data-sharing/
When available: With publication

Supporting Documents
Document types: None

Additional Information
Who can access the data: Researchers whose proposed use of the data has been approved
Types of analyses: Any purpose
Mechanisms of data availability: With investigator support
